# Geographic Differentiation of Essential Oil from Rhizome of Cultivated *Atractylodes lancea* by Using GC-MS and Chemical Pattern Recognition Analysis

**DOI:** 10.3390/molecules28052216

**Published:** 2023-02-27

**Authors:** Baohong Song, Wei Wang, Ruipeng Liu, Jinjin Cai, Yuanyuan Jiang, Xuemei Tang, Hongfei Wu, Hui Ao, Lu Chen

**Affiliations:** 1 State Key Laboratory of Southwestern Chinese Medicine Resources, School of Pharmacy, Chengdu University of Traditional Chinese Medicine, Chengdu 611137, China; 2 Hospital of Chengdu University of Traditional Chinese Medicine, Chengdu 610075, China; 3 Chengdu Institute of Food Inspection, Chengdu 610045, China; 4 Innovative Institute of Chinese Medicine and Pharmacy, Chengdu University of Traditional Chinese Medicine, Chengdu 611137, China

**Keywords:** *Atractylodes lancea*, GC-MS, essential oil, chemical pattern recognition analysis, geographic origin

## Abstract

The rhizome of *Atractylodes lancea* (RAL) is a well-known Chinese herbal medicine (CHM) that has been applied in clinical settings for thousands of years. In the past two decades, cultivated RAL has gradually replaced wild RAL and become mainstream in clinical practice. The quality of CHM is significantly influenced by its geographical origin. To date, limited studies have compared the composition of cultivated RAL from different geographical origins. As essential oil is the primary active component of RAL, a strategy combining gas chromatography-mass spectrometry (GC-MS) and chemical pattern recognition was first applied to compare the essential oil of RAL (RALO) from different regions in China. Total ion chromatography (TIC) revealed that RALO from different origins had a similar composition; however, the relative content of the main compounds varied significantly. In addition, 26 samples obtained from various regions were divided into three categories by hierarchical cluster analysis (HCA) and principal component analysis (PCA). Combined with the geographical location and chemical composition analysis, the producing regions of RAL were classified into three areas. The main compounds of RALO vary depending on the production areas. Furthermore, a one-way analysis of variance (ANOVA) revealed that there were significant differences in six compounds, including modephene, caryophyllene, γ-elemene, atractylon, hinesol, and atractylodin, between the three areas. Hinesol, atractylon, and β-eudesmol were selected as the potential markers for distinguishing different areas by orthogonal partial least squares discriminant analysis (OPLS-DA). In conclusion, by combining GC-MS with chemical pattern recognition analysis, this research has identified the chemical variations across various producing areas and developed an effective method for geographic origin tracking of cultivated RAL based on essential oils.

## 1. Introduction

**Figure 1:**
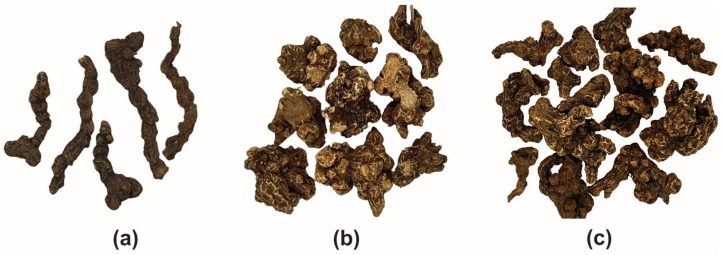
Wild and cultivated RAL. (**a**) Wild RAL; (**b**) cultivated RAL (Mao Mountain); (**c**) cultivated RAL (Yingshan).

The rhizome of *Atractylodes lancea* (RAL), also called maocangzhu or nancangzhu, is a well-known traditional Chinese medicine (TCM), which has been used clinically for thousands of years. RAL was first recorded in the Shen Nong Ben Cao Jing (Han Dynasties) and is traditionally used to treat conditions, such as rheumatic diseases, digestive disorders, night blindness, and influenza [1,2]. Prior to 2000, RAL production was dominated by wild resources. Mao Mountain in Jiangsu province was traditionally considered a genuine producing area, and RAL from Mao Mountain was believed to be of the best quality. However, owing to environmental changes and the scarcity of wild RAL resources, cultivated RAL has gradually become mainstream in the past 20 years, and wild RAL has rarely been found in Chinese markets for medicinal materials. In addition, the Hubei, Anhui, Shaanxi, and Henan Provinces are the main producing areas of RAL at present [3,4]. As presented in Figure 1, as the production mode changed, the appearance of RAL also changed from a continuous bead-shaped cylinder (wild RAL) to a lump-shaped or irregular-shaped cylinder (cultivated RAL).

Geographic origin is a well-known important factor affecting the quality of Chinese herbal medicines (CHMs) [5]. Medicinal materials from different producing areas often have significant differences in chemical composition and clinical efficacy. It is crucial to distinguish CHMs with different geographical origins. Although there have been some relevant studies on the essential oil of wild RAL from various habitats [6,7], no geographic traceability study has been performed on cultivated RAL, which has dominated the TCM markets in the past decade. This greatly limits the quality evaluation of RAL and is not conducive to the development and application of RAL.

RAL is abundant in essential oil. The essential oil of RAL (RALO) mainly contains sesquiterpenes and polyacetylenes, among which hinesol, atractylon, β-eudesmol, and atractydin are the most abundant and widely reported [8]. These compounds have a variety of pharmacological effects, including antitumor [9,10], antiinflammatory, analgesic [11], antiviral [12] and gastrointestinal-protective [13,14] activities, etc. In addition, RALO has been proven to promote mesenchymal stem cells (MSCs) chondrogenic differentiation [15] and inhibit the growth of *Helicobacter pylori* (HP) in a concentration-dependent manner [16], which is closely related to the traditional use of RAL to treat osteoarthritis and gastrointestinal disorders. Therefore, the essential oil is a crucial index of the quality of RAL. It is a wise choice to select appropriate technical strategies to compare cultivated RAL from different geographical origins and explore its geographic differentiation based on essential oil.

Owing to the high complexity and variability of the chemical composition of TCM [17], it is difficult to distinguish samples from different areas by routine intuitive comparison of chemical composition. Gas chromatography-mass spectrometry (GC-MS) is frequently employed in the analysis and identification of volatile substances, due to its high sensitivity, performance, and stability [18]. The fingerprint has the advantage of integrity. It can simultaneously characterize the complex components of CHMs and provide a wealth of identification information, which has been accepted as a method to access the quality of traditional medicine by the World Health Organization (WHO) [19,20]. Chemical pattern-recognition analysis is a multivariate analysis technique that can reveal the law behind the measured data and that demonstrates significant advantages in differentiating samples through the analysis and visualisation of high-dimensional data [21]. In recent years, chemometric methods such as hierarchical cluster analysis (HCA), principal component analysis (PCA), and orthogonal partial least squares discriminant analysis (OPLS-DA) has been integrated with chemical analysis, widely used in the identification, evaluation, and characterization of traditional medicines, food, spices, etc. [22,23,24,25,26]. Therefore, it may be a useful tool with which to solve the aforementioned problems.

Overall, this study combined GC-MS and chemical pattern-recognition analysis and compared the essential oil of cultivated RAL from the main producing regions in China. Specifically, GC-MS fingerprints and chemometric methods, including HCA and PCA, were used to distinguish and classify different regions. Moreover, one way analysis of variance (ANOVA) and OPLS-DA were utilised to identify the potential chemical markers for differentiating between different producing areas. This article aims to investigate the chemical differences of essential oil in cultivated RAL from different regions and provide an effective method by which to trace the geographic origin of cultivated RAL.

## 2. Results and Discussion

### 2.1. Fingerprints of RAL

**Figure 2:**
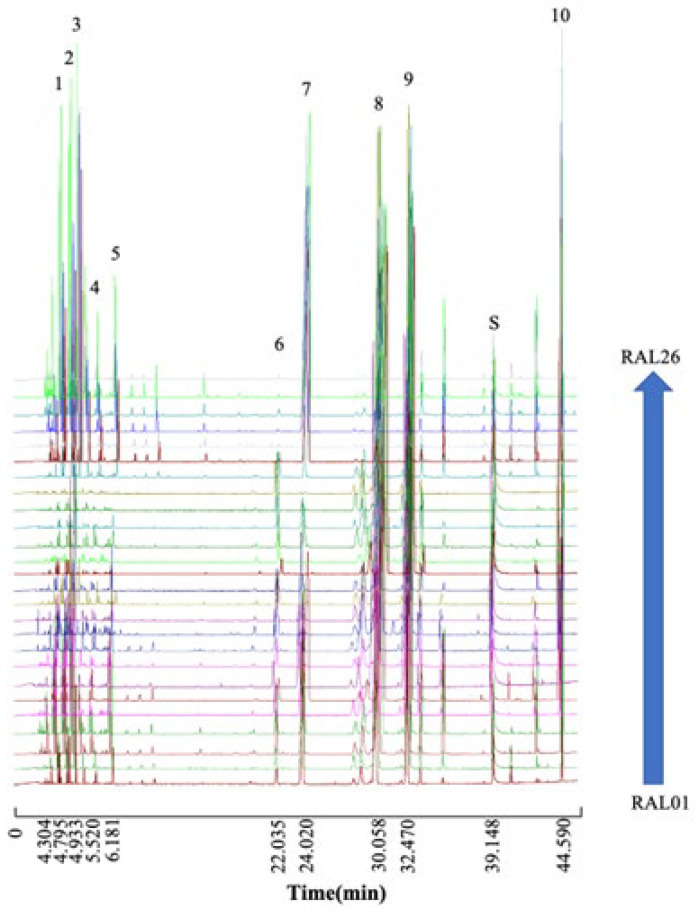
GC-MS chromatogram of 26 batches of RAL from different regions. (1) α-guaiene; (2) modephene; (3) berkheyaradulene; (4) caryophyllene; (5) γ-elemene; (6) elemol; (7) atractylon; (8) hinesol; (9) β-eudesmol; (S) ISTD: 1-hexadecanol; (10) atractylodin.

Total iron chromatography (TIC) reveals that the chemical compositions of the 26 RAL samples were generally similar. A total of 10 characteristic shared peaks were found from the fingerprint profile, which was shown in Figure 2. The sum of their peak areas exceeded 70% of the total area in all samples. This suggests that the 10 peaks could represent the chemical features of RAL relatively well. According to the NIST14 database of the National Institute of Standards and Technology, peaks 1 to 10 were identified as α-guaiene, modephene, berkheyaradulene, caryophyllene, γ-elemene, elemol, atractylon, hinesol, β-eudesmol, and atractylodin, with the matching degree all above 90%, as demonstrated in Supplementary Table S1.

Although the fingerprints of RAL from different regions have 10 peaks in common, there are still many differences. The fingerprint similarities of sample RAL01–RAL20 ranged from 0.926–0.998, whereas RAL21–RAL26 were all below 0.800. In particular, the samples from Mao Mountain were different from those from other places. It could be seen from the TIC that the peak areas of samples from Mao Mountain were significantly larger than those from other regions at 4–7 min (peak 1, 2, 3, 4, and 5), 23–25 min (peak 7), and 43–45 min (peak 10), whereas at 25–33 min, the peak areas of other regions were higher than those of Mao Mountain (peak 8 and 9). According to the previous studies, the most notable chemical compounds of RALO are as follows: atractylodin, atractylon, hinesol and β-eudesmol [27,28]. As observed from Table S1, the samples with high relative contents of atractylon (>30%) and atractylodin (>10%) were all collected from Mao Mountain. Almost all the samples with a relative content of >40% of hinesol were obtained from Shangluo (Shaanxi Province), Nanyang (Henan Province), and Shiyan (Hubei Province), which share a close geographical location. The relative contents of β-eudesmol in RALO from other origins, except for those from Mao Mountain (<15.5%), ranged from 21% to 37%.

### 2.2. HCA

**Figure 3:**
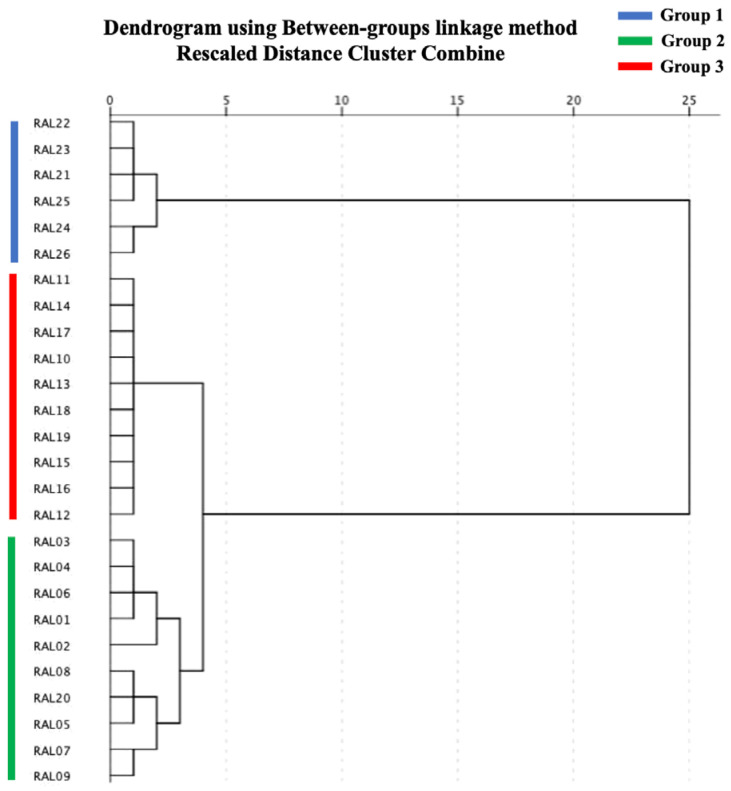
HCA dendrogram of 26 RAL samples from different regions using the between-groups linkage method based on squared Euclidean distance.

For classified groups of samples, it was common to use HCA, an unsupervised pattern-recognition method, and the degree of similarity depends on the characteristics of the variables [29]. To further classify and compare RALO from different geographical origins and reveal the influence of geographical origin on chemical composition, the relative contents of the 10 characteristic compounds of RALO were imported into SPSS software version 26.0 by using the intergroup connection method and the Euclidean square distance as the metric to perform clustering. Figure 3 depicts the finding of the analysis. Twenty-six RAL samples from different origins were divided into three groups when the classification distance was 4, which is as follows: Group 1 (RAL21–RAL26) was all collected in Mao Mountain, Jiangsu Province; Group 2 (RAL01–RAL09, RAL20) except RAL20 (obtained from Shangluo, Shaanxi Province), were produced in Huanggang (Hubei Province), Anqing (Anhui Province), Lu’an (Anhui Province) and Xinyang (Henan Province). Although these producing areas are in different provinces, they are geographically close. They all from Dabie Mountains where wild *Atractylodes lancea* was once abundant. Group 3 (RAL10–RAL19) was produced in Shiyan (Hubei Province), Shangluo (Shaanxi Province), and Nanyang (Henan Province), which are geographically close and belong to the Qin-ba Mountains.

**Figure 4:**
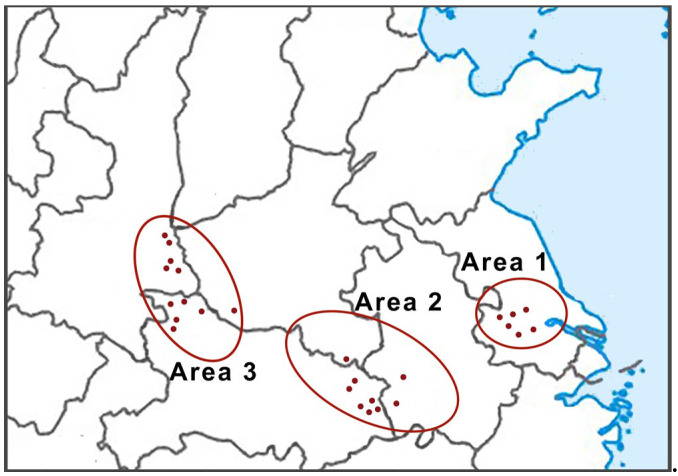
Twenty-six batches of RAL collection regions in China.

This demonstrates that the chemical constituents of the essential oil of cultivated RAL from different geographic origins differ and that chemical compositions from similar producing regions are often similar. The HCA model can effectively distinguish samples from different producing areas based on these 10 characteristic compounds of the RALO. Combined with geographical location and HCA, the origin of RAL was divided into three producing areas (Figure 4). Area 1 was Mao Mountain (Jiangsu Province), which is the traditional authentic area of production of RAL. Area 2 was the Dabie Mountains, including Huanggang (Hubei Province), Anqing (Anhui Province), Lu’an (Anhui Province) and Xinyang (Henan Province), which is the main producing area of cultured RAL at present. Area 3 was Qin-ba Mountains, including Shangluo (Shaanxi Province), Nanyang (Henan Province), and Shiyan (Hubei Province), which is also a new producing area for cultivated RAL in recent years. Moreover, the samples from Area 2 and Area 3 were first grouped together and further with the samples from Area 1, which revealed that the samples from Dabie Mountains and Qin-ba Mountains possessed more similarity than those from Mao Mountain.

### 2.3. PCA

**Figure 5:**
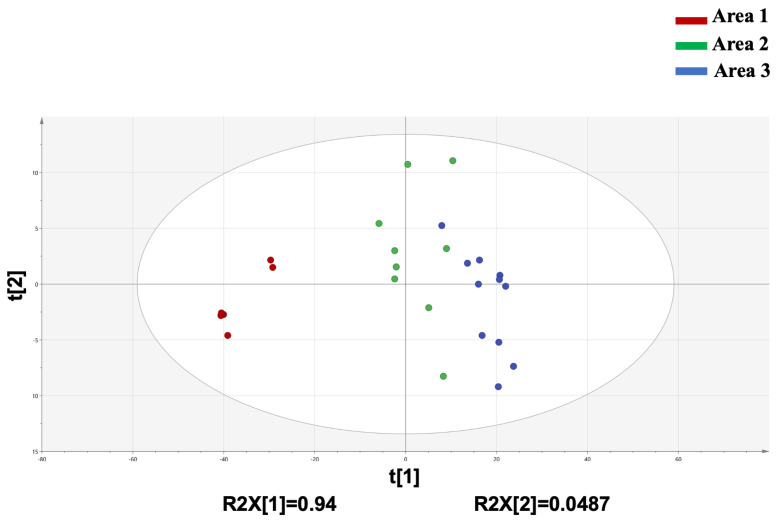
Score plot of PCA of 26 samples of RAL from different regions.

PCA is an unsupervised pattern recognition technology that uses linear dimension reduction to cluster and visualize high-dimensional data [30,31]. PCA was performed based on the 10 characteristic components to provide more information on the differentiation of the geographical origins of RAL samples. The relative contents of 10 characteristic compounds were regarded as the variables in the PCA model after the initial data centering. Two principal components (PCs) were extracted with eigenvalues >1.0, which explained 98.87% (PC1, 94.0%; PC2, 4.87%) of the original 10 variables. The Q2 value was 0.943, representing a good predictive power of this model. As presented in Figure 5, the 26 RAL samples from different producing areas were roughly divided into three groups. The dots of Area 2 and Area 3 were relatively dense, indicated that the RALO of the two areas shared more similarity than that of Area 1. This result was consistent with that of HCA.

### 2.4. Chemical Composition Analysis and ANOVA

**Figure 6:**
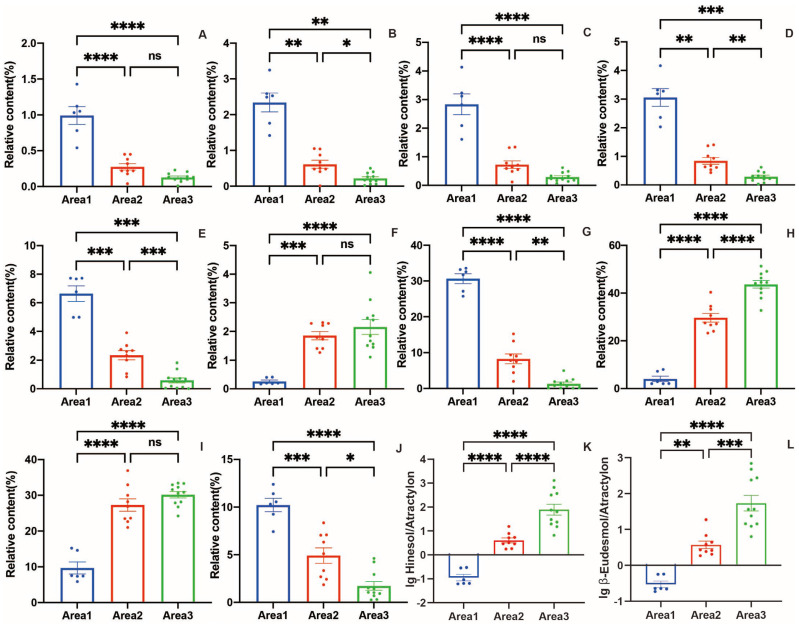
Relative contents of the characteristic composition in total ion chromatography (TIC) of area 1, area 2, and area 3. (**A**) α-guaiene; (**B**) modephene; (**C**) berkheyaradulene; (**D**) caryophyllene; (**E**) γ-elemene; (**F**) elemol; (**G**) atractylon; (**H**) hinesol; (**I**) β-eudesmol; (**J**) atractylodin; (**K**) lg hinesol/atractylon; (**L**) lg β-eudesmol/atractylon. Data were shown as mean ± SEM. **** *p* < 0.0001, *** *p* < 0.001, ** *p* < 0.01, * *p* < 0.05, ns was considered as no significant difference.

Based on these results, the relative contents of 10 common peaks of RALO from Area 1, Area 2, and Area 3 were compared. As presented in Supplementary Table S1 and Figure 6, ANOVA was used to analyse the relative contents of the 10 characteristic compounds of RAL. The P-value was set as the filtering standard to maintain the contents. The relative contents of the 10 characteristic compounds differed in different areas. First, the main components of the essential oil of the samples from Area 1 were atractylon and atractylodin, with average relative contents being 30.66 ± 3.07% and 10.22 ± 1.58%, respectively. The main components of the essential oil of the samples from Area 2 and Area 3 were hinesol and β-eudesmol. In addition, the samples from Area 3 had the highest average relative contents of hinesol (43.70 ± 5.10%) and β-eudesmol (30.19 ± 2.88%). The samples from Area 2 were similar to those from Area 3; however, the average relative contents of hinesol (29.67 ± 5.22%) and β-eudesmol (27.30 ± 4.97%) were relatively low. Moreover, the relative contents of five compounds—modephene, caryophyllene, γ-elemene, atractylon and atractylodin—from RALO of Area 1 were significantly higher than those from Area 2, which were further significantly higher than those from Area 3. The relative contents of elemol of Area 2 (1.85 ± 0.40%) and Area 3 (2.16 ± 0.83%) were significantly higher than those of Area 1 (0.26 ± 0.10%). In a word, the chemical composition of RALO from different producing areas showed obvious geographical differentiation.

Additionally, the ratios between the relative contents of principal components were further explored. The ratios of hinesol to atractylon (H/A) and β-eudesmol to atractylon (E/A) were found to be of high distinguishing value. As shown in Supplementary Table S1, H/A and E/A of the samples from Area 1 were all less than 1. In Area 2, both ratios were ranged from 1 to 10 (except RAL05). In area 3, they were greater than 10 (except RAL20). The average of H/A in the three producing areas were 0.14, 5.16, and 260.47 respectively. The average of E/A in the three producing areas were 0.33, 5.03, and 166.39 individually. The bar charts were drawn with the common logarithm of the ratios as the ordinates, as presented in Figure 6K,L. These two ratios (H/A, E/A) and their common logarithms can be used to achieve geographic origin tracking of cultivated RAL conveniently.

Some studies have been conducted on the geographic variation of essential oil in wild RAL; however, the results are quite different, and there are many controversies [32,33,34]. Our study demonstrated that the RAL of Mao Mountain was significantly different from that of other regions, and the RAL of the Dabie Mountains in Hubei Province was also significantly different from that of areas outside the Dabie Mountains in Hubei Province. RALO can be divided into three chemical types depending on geographical origin, which was consistent with the findings of Takeda, a Japanese scholar [7,35]. Ancient Chinese medicine books have considered Mao Mountain in Jiangsu province an authentic RAL-producing area, suggesting that the RAL produced there is of relatively better efficacy. Although the wild RAL with good quality has been replaced by the cultivated RAL in recent years, this study reveals that the chemical composition of cultivated RAL from Mao Mountain remains significantly different from that of other producing areas. Studies have reported that the contents of hinesol and β-eudesmol decreased significantly in transplantation to Mao Mountain [27,36]. This may be attributed to the higher average annual temperature, abundant rainfall, and short dry season of Mao Mountain [37]. Notably, whether its efficacy is obviously better than that of other areas is a topic worthy of further study. Still, this research strongly proves the wisdom of emphasising the producing regions of medicinal materials in traditional Chinese medicine.

### 2.5. OPLS-DA

**Figure 7:**
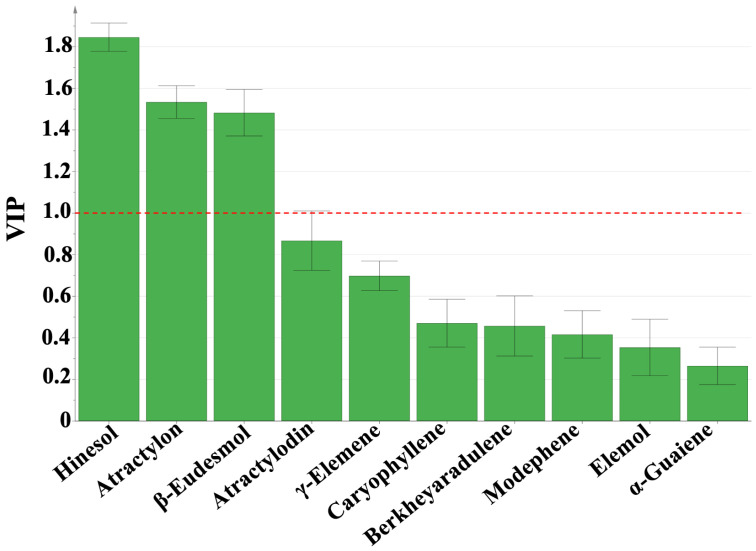
VIP values of the 10 characteristic compounds of 26 samples of RAL from different regions based on OPLS-DA.

Although both HCA and PCA can obtain origin distinctions, the effect of one variable on sample classification cannot be elucidated by the two identification methods. To further investigate the differentiation of RAL in different regions and identify important variables (key markers) for geographic origin tracking, a supervised OPLS-DA technique was used to analyze the relative contents of 10 characteristic components. Variable importance in the projection (VIP) values were used to evaluate the amount that each component contributed to group separation. In this research, compounds with VIP values > 1 were selected as the key markers for sample classification. The R2X, R2Y, and Q2 (cum) values of the OPLS-DA model were 0.967, 0.556, and 0.495, respectively, indicating the good fitting and prediction ability of the model. Figure 7 represents the ten characteristic compounds sorted by VIP value in descending order. Three compounds with VIP > 1 were discovered, namely hinesol, atractylon, and β-eudesmol, which were considered important chemical markers to distinguish the origins. It is worth noting that hinesol, atractylon, and β-eudesmol were the essential compounds with the highest content in RAL, which also exhibited excellent value in discriminating RAL from different areas using ANOVA. Atractylon has antitumor [10], antiinflammatory, antinociceptive [11], antiviral [12], and neuroprotective activities [38]. Hinesol possesses antitumor [9] and antigastric ulcer [14] effects. β-eudesmol can regulate gastrointestinal function [39], suppress tumour growth [40], and inhibit angiogenesis [13]. Considering the rich pharmacological activity of these compounds, the differentiation in biological activity and clinical efficacy of RAL from different regions must be investigated further.

## 3. Materials and Methods

### 3.1. Plants Materials

RAL01–RAL20 were collected from the cultivation region of Hubei, Anhui, Shaanxi, and Henan Provinces in China. RAL21–RAL26 were purchased from Jiangsu Maoshan TCM Planting Co., Ltd. All samples were deposited in the State Key Laboratory of Southwestern Chinese Medicine Resources (Chengdu University of TCM), and the sample information is presented in Table 1.

### 3.2. Solvents and Chemicals

All analytical grades of hexane (lot 211164) were produced by Thermo Fisher Scientific (China) Co., Ltd. (Shanghai, China), sodium sulfate (lot 2020091401) was provided by Chengdu Kelong Chemical Co., Ltd. (Chengdu, China), and 1-hexadecanol (lot C2116115) was provided by Shanghai Aladdin Biochemical Technology Co., Ltd. (Shanghai, China). Other reagents were of all analytical grades.

### 3.3. Extraction of Volatile Oil and Preparation of Test Products

Approximately 50.0 g of RAL was crushed and put into a 1000-mL round bottom flask. Ten times as much distilled water was added, mixed, and placed in an electric heating cap. The mixture was slowly heated to boiling and simmered for about 5 h until the amount of volatile oil in the tester did not increase. The volatile oil was collected in a brown bottle, dried by adding a small amount of anhydrous sodium sulfate, sealed, and stored in a refrigerator at 4 °C. The internal standard solution of 6.0 mg·mL^−1^ was obtained by precisely weighing 60.0 mg of 1-hexadecanol and dissolving it in 10 mL of hexane. Thirty microliters of the obtained essential oil were dissolved in 1.5 mL hexane and 90 μL of internal standard solution was added. Furthermore, the mixture was filtered through a 0.22 μM filter, and the filtrate was used as a GC-MS testing solution.

### 3.4. GC-MS Analysis Conditions

Referring to the previous study [41], we optimized the analysis conditions as follows. GC-MS analysis of the RALO was performed by using Agilent GC–MS (7890A-5975C, Agilent, Santa Clara, CA, USA) with an MS capillary column (HP-5, Agilent,, Santa Clara, CA, USA, 30 m × 0.25 mm × 0.25 µm). The injection volume was 1 µL with a 100:1 (*v*/*v*) ratio split mode. The carrier gas was helium (99.999% purity, 1 mL·min^−1^). The temperature of the injector and interface was set to 250 °C and 280 °C, respectively. A programmed temperature rise was adopted with an initial column temperature of 100 °C, rising to 135 °C at 10 °C·min^−1^, then to 145 °C at 0.5 °C·min^−1^ and holding for 6 min, and to 250 °C at 5 °C·min^−1^ and holding for 8 min. The mass spectrometer was operated at 70 ev in full scan mode. The compounds in RALO were identified through the NIST14 database. The area normalisation method calculated the relative content of each compound in the chromatogram.

### 3.5. Data Analysis

GC fingerprint similarity was evaluated by using the “Chromatographic Fingerprint Similarity Evaluation System for Traditional Chinese Medicine” (2012 version). Statistical analysis was performed by using the one-way ANOVA by GraphPad Prism 9 (GraphPad Software Inc., La Jolla, CA, USA). In addition, these data were analysed and processed by HCA by using SPSS26.0 (SPSS Inc., Chicago, IL, USA). PCA and OPLS-DA using SIMCA P14.1 (Umetrics, Umea, Sweden). The results were expressed in means ± standard error of mean (SEM), and the level of *p* < 0.05 was considered statistically significant.

## 4. Conclusions

In this study, an efficient method by which to trace the geography of cultivated RAL was developed by GC-MS and chemical pattern recognition analysis. The GC-MS fingerprints revealed that the RAL cultivated in different regions were similar and observed a total of 10 common peaks. The difference was also significant. Combining PCA and HCA, the producing regions of RAL can be divided into three areas (Figure 4). Area 1 is Mao Mountain (Jiangsu Province), which is the traditional genuine producing area of RAL. Area 2 is the Dabie Mountains, including Huanggang (Hubei Province), Anqing (Anhui Province), Xinyang (Henan Province), and Lu’an (Anhui Province), which is currently the main producing area of cultured RAL. Area 3 is the Qin-ba Mountains, including Shangluo (Shaanxi Province), Nanyang (Henan Province), and Shiyan (Hubei Province), which is also a new producing area for cultivated RAL in recent years.

According to the analysis of the relative contents of characteristic compounds, it was discovered that the primary components of the volatile oil of RAL varied from different producing areas. The primary compounds of RALO from Area 1 were atractylon and atractylodin, whereas the primary compounds of RALO from Area 2 and Area 3 were hinesol and β-eudesmol. Furthermore, there were significant differences in the relative contents of the samples from Area 2 and Area 3. By using ANOVA, it was observed that the contents of six compounds, including hinesol, modephene, caryophyllene, γ-elemene, atractylon, and atractylodin, differed significantly between the three areas by ANOVA. In addition, three compounds with VIP > 1 including hinesol, atractylon, and β-eudesmol were discovered by OPLS-DA and may serve as important chemical markers to distinguish the samples from different producing areas.

In conclusion, this research identified the chemical variations of cultivated RAL from different producing areas and developed an effective approach for tracing their geographic origin based on essential oils by combining GC-MS with chemical pattern recognition analysis. Hinesol, atractylon, and β-eudesmol can be used as chemical markers to distinguish between different areas. The RAL essential oil cultivated in the traditional genuine producing area was significantly different from the other two main producing areas. The essential oil of cultivated RAL from the Dabie Mountains and Qin-ba Mountains was also different. This paper can provide references for other similar studies.

## Figures and Tables

**Table 1:** Sample information of 26 batches of RAL.

NO.	Place of Origin	Sampling Time	Growth Years	Batch Code
RAL01	Yingshan, Huanggang, Hubei	2021.11	3	2021M001
RAL02	Yingshan, Huanggang, Hubei	2021.11	3	2021M002
RAL03	Yingshan, Huanggang, Hubei	2021.11	3	2021M003
RAL04	Yingshan, Huanggang, Hubei	2021.11	3	2021M004
RAL05	Luotian, Huanggang, Hubei	2021.11	3	2021M005
RAL06	Luotian, Huanggang, Hubei	2021.11	3	2021M006
RAL07	Shihe, Xinyang, Henan	2021.11	3	2021M007
RAL08	Huoshan, Lu’an, Anhui	2021.11	3	2021M008
RAL09	Yuexi, Anqing, Anhui	2021.11	3	2021M009
RAL10	Fangxian, Shiyan, Hubei	2021.11	3	2021M010
RAL11	Fangxian, Shiyan, Hubei	2021.11	3	2021M011
RAL12	Yunxi, Shiyan, Hubei	2021.12	3	2021M012
RAL13	Yunxi, Shiyan, Hubei	2021.12	3	2021M013
RAL14	Yunyang, Shiyan, Hubei	2021.12	3	2021M014
RAL15	Dengzhou, Nanyang, Henan	2021.12	3	2021M015
RAL16	Luonan, Shangluo, Shaanxi	2021.11	3	2021M016
RAL17	Luonan, Shangluo, Shaanxi	2021.11	3	2021M017
RAL18	Danfeng, Shangluo, Shaanxi	2021.11	3	2021M018
RAL19	Danfeng, Shangluo, Shaanxi	2021.11	3	2021M019
RAL20	Danfeng, Shangluo, Shaanxi	2021.11	3	2021M020
RAL21	Mao Mountain, Jiangsu	2021.11	3	2021M021
RAL22	Mao Mountain, Jiangsu	2021.11	3	2021M022
RAL23	Mao Mountain, Jiangsu	2021.11	3	2021M023
RAL24	Mao Mountain, Jiangsu	2021.11	3	2021M024
RAL25	Mao Mountain, Jiangsu	2021.11	3	2021M025
RAL26	Mao Mountain, Jiangsu	2021.11	3	2021M026

## Data Availability

Data are contained within the article.

## Supplementary Materials

The following supporting information can be downloaded at: https://www.mdpi.com/article/10.3390/molecules28052216/s1. Table S1: Relative contents (%) of characteristic components of 26 batches of RAL from different regions.

## Sample Availability

Samples of the compounds are not available from the authors.
