# Supplementary material for: Geographic Differentiation of Essential Oil from Rhizome of Cultivated Atractylodes lancea by Using GC-MS and Chemical Pattern Recognition Analysis
Source: Molecules. 2023 Feb 27;28(5):2216. doi: 10.3390/molecules28052216 (PMC10004716; doi:10.3390/molecules28052216)
Supplement: Supplementary file 1 [file molecules-28-02216-s001.zip › molecules-2180092-SI.pdf]

Table S1. Relative contents (%) of characteristic components of 26 batches of RAL from different regions.

NO.	Retention Time	Component	Matching Degree	Molecular Formula	RAL 01	RAL 02	RAL 03	RAL 04	RAL 05	RAL 06	RAL 07	RAL 08	RAL 09	RAL 10	RAL 11	RAL 12	RAL 13	RAL 14	RAL 15	RAL 16	RAL 17	RAL 18	RAL 19	RAL 20	RAL 21	RAL 22	RAL 23	RAL 24	RAL 25	RAL 26	Average relative contents in area 1	Average relative contents in area 2	Average relative contents in area 3
1	4.304	α-Guaiene	96%	$C_{15}H_{24}$	0.38	0.22	0.45	0.45	0.32	0.04	0.18	0.22	0.22	0.14	0.18	0.21	0.13	0.11	0.10	0.13	0.10	0.01	0.07	0.23	1.12	1.03	1.05	0.78	1.43	0.54	0.99±0.28	0.28±0.13	0.13±0.06
2	4.795	Modephene	99%	$C_{15}H_{24}$	0.87	0.48	1.05	1.04	0.67	0.01	0.41	0.5	0.48	0.27	0.40	0.37	0.26	0.02	0.06	0.17	0.14	0.15	0.06	0.50	2.59	2.49	2.53	1.76	3.25	1.42	2.34±0.60	0.61±0.32	0.22±0.15
3	4.933	Berkheyaradulene	94%	$C_{15}H_{24}$	0.71	0.59	1.32	1.34	0.78	0.12	0.49	0.63	0.55	0.32	0.49	0.45	0.33	0.23	0.14	0.21	0.18	0.17	0.07	0.62	3.23	3.12	2.82	2.09	4.13	1.61	2.83±0.81	0.73±0.37	0.29±0.16
4	5.520	Caryophyllene	99%	$C_{15}H_{24}$	0.93	0.63	1.37	1.40	1.01	0.43	0.53	0.67	0.61	0.31	0.48	0.44	0.32	0.30	0.03	0.22	0.20	0.16	0.07	0.62	3.32	3.19	3.28	2.36	4.17	2.03	3.06±0.70	0.84±0.34	0.29±0.17
5	6.181	γ-Elemene	98%	$C_{15}H_{24}$	2.61	2.75	2.32	2.67	0.84	3.90	2.00	1.05	3.01	0.04	0.76	0.68	0.68	0.01	0.11	1.39	0.72	0.18	0.05	1.81	7.67	7.72	7.72	4.98	6.77	4.99	6.64±1.22	2.35±0.90	0.58±0.57
6	22.035	Elemol	97%	$C_{15}H_{26}O$	1.41	1.41	2.28	2.28	2.31	1.27	1.73	1.79	2.21	3.11	2.55	1.45	1.51	1.11	1.84	1.67	2.47	4.06	2.40	1.56	0.20	0.18	0.20	0.41	0.17	0.40	0.26±0.10	1.85±0.40	2.16±0.83
7	24.020	Atractylon	99%	$C_{15}H_{20}O$	15.23	7.47	9.06	9.37	1.98	13.21	6.12	4.43	7.80	0.14	1.87	0.77	1.35	0.07	0.04	2.13	2.51	0.66	0.12	4.99	32.58	33.59	33.20	27.14	31.76	25.74	30.66±3.07	8.30±3.87	1.33±1.43
8	30.058	Hinesol	93%	$C_{15}H_{26}O$	27.49	24.03	26.61	27.06	30.62	23.22	33.19	34.49	40.35	44.55	44.12	48.08	40.14	46.33	51.31	41.89	37.90	49.15	44.49	32.7	2.09	2.06	2.12	8.04	2.93	7.28	4.08±2.55	29.67±5.22	43.70±5.10
9	32.470	β-Eudesmol	99%	$C_{15}H_{26}O$	28.21	32.96	23.61	22.66	36.92	26.87	23.47	30.03	21.01	33.41	26.78	28.15	32.30	33.31	27.87	30.58	30.85	24.25	32.94	31.63	7.35	7.40	7.50	15.23	5.89	14.63	9.67±3.76	27.30±4.97	30.19±2.88
S	39.148	1-Hexadecanol	99%	$C_{16}H_{34}O$	3.45	3.73	2.86	2.21	4.94	2.06	4.43	5.24	2.93	1.69	5.95	2.31	6.13	4.33	3.67	2.30	5.50	6.84	8.78	4.38	2.50	3.26	2.22	2.02	2.41	3.19	2.60±0.47	3.54±1.08	4.72±2.07
10	44.590	Atractylodin	93%	$C_{13}H_{10}O$	2.43	6.33	7.05	7.09	3.34	8.36	1.86	5.12	2.69	1.03	1.42	2.93	1.53	1.06	0.27	4.26	0.93	0.71	0.31	4.62	10.30	10.56	11.40	9.26	7.43	12.39	10.22±1.58	4.92±2.26	1.73±1.45
		Total			83.72	80.6	77.98	77.57	83.73	79.49	74.41	84.17	81.86	85.01	85.00	85.84	84.68	86.88	85.44	84.95	81.50	86.34	89.36	83.66	72.95	74.60	74.04	74.07	70.34	74.22	73.37±1.45	80.39±3.15	85.33±1.86
		Hinesol/Atractylon			1.801	3.22	2.94	2.89	15.47	1.76	5.42	7.79	5.17	318.21	23.59	62.44	29.73	661.86	1282.75	19.67	15.10	74.47	370.75	6.55	0.06	0.06	0.06	0.30	0.09	0.28	0.14±0.10	5.16±4.08	260.47±379.89
		β-Eudesmol/Atractylon			1.85	4.41	2.61	2.418	18.65	2.03	3.84	6.78	2.69	238.64	14.32	36.56	23.93	475.86	696.75	14.36	12.29	36.74	274.50	6.34	0.23	0.22	0.23	0.56	0.19	0.57	0.33±0.17	5.03±5.03	166.39±222.29
